# When Neuroscience Meets Pharmacology: A Neuropharmacology Literature Analysis

**DOI:** 10.3389/fnins.2018.00852

**Published:** 2018-11-16

**Authors:** Andy Wai Kan Yeung, Nikolay T. Tzvetkov, Atanas G. Atanasov

**Affiliations:** ^1^Oral and Maxillofacial Radiology, Applied Oral Sciences, Faculty of Dentistry, The University of Hong Kong, Hong Kong, China; ^2^Department of Biochemical Pharmacology and Drug Design, Institute of Molecular Biology “Roumen Tsanev”, Bulgarian Academy of Sciences, Sofia, Bulgaria; ^3^Pharmaceutical Institute, University of Bonn, Bonn, Germany; ^4^The Institute of Genetics and Animal Breeding, Polish Academy of Sciences, Magdalenka, Poland; ^5^Department of Pharmacognosy, University of Vienna, Vienna, Austria; ^6^GLOBE Program Association (GLOBE-PA), Grandville, MI, United States

**Keywords:** Alzheimer's disease, bibliometrics, compounds, drugs, molecules, neuropharmacology

## Abstract

**Background:** Considering the enormous progress in the field of neuropharmacology and its global importance, as well as the lack of bibliometric studies examining this field as a whole, it is a high time to assess the prevailing topics and citation performances of its research works.

**Methods**: Web of Science (WoS) was searched to identify relevant neuropharmacology articles, which were analyzed with reference to (1) publication year, (2) journal title, (3) total citation count, (4) authorship, (5) WoS category, and (6) manuscript type. The identified manuscripts were analyzed with VOSviewer for further bibliometric parameters, such as citation analysis of institutions, countries/regions, and journals, and to visualize the citation patterns of the terms appearing in the titles and abstracts.

**Results**: The literature search resulted in 43,354 manuscripts. Nearly 98% of them were published since the 1990s. The majority of the manuscripts were original articles (*n* = 31,360) and reviews (*n* = 11,266). The top five WoS categories associated with the analyzed manuscripts were Pharmacology/Pharmacy (*n* = 14,892, 34.3%), Neurosciences (*n* = 11,747, 27.1%), Clinical Neurology (*n* = 4,981, 11.5%), Psychiatry (*n* = 4,464, 10.3%), and Biochemistry/Molecular Biology (*n* = 4,337, 10.0%). Seven of the top ten most prolific institutions were located in the USA, and one each in Canada, Italy, and the UK, respectively. Manuscripts mentioning certain molecules or pharmaceuticals had high citations per manuscript, such as those reporting about anandamide, tetrahydrocannabinol (THC), L-glutamate, clozapine, and curcumin. These terms with at least 50 citations per manuscript were mostly related to cannabis and anti-psychotic drugs, with some dealing with anti-epilepsy effects and Alzheimer's disease.

**Conclusion**: We have identified and analyzed all neuropharmacology articles published since the 1990s. Importantly, the area of neuropharmacology research has been growing steadily due to the global trend in population aging and associated with this continuously increasing number of patients with neuropsychiatric disorders worldwide. It is hoped that identification of new pharmaceutically useful molecules or new clinical applications will continue in the future, in order to improve clinical outcomes and to further strengthen the field of neuropharmacology, a research area cross-linking basic and clinical sciences.

## Introduction

**Figure 1:**
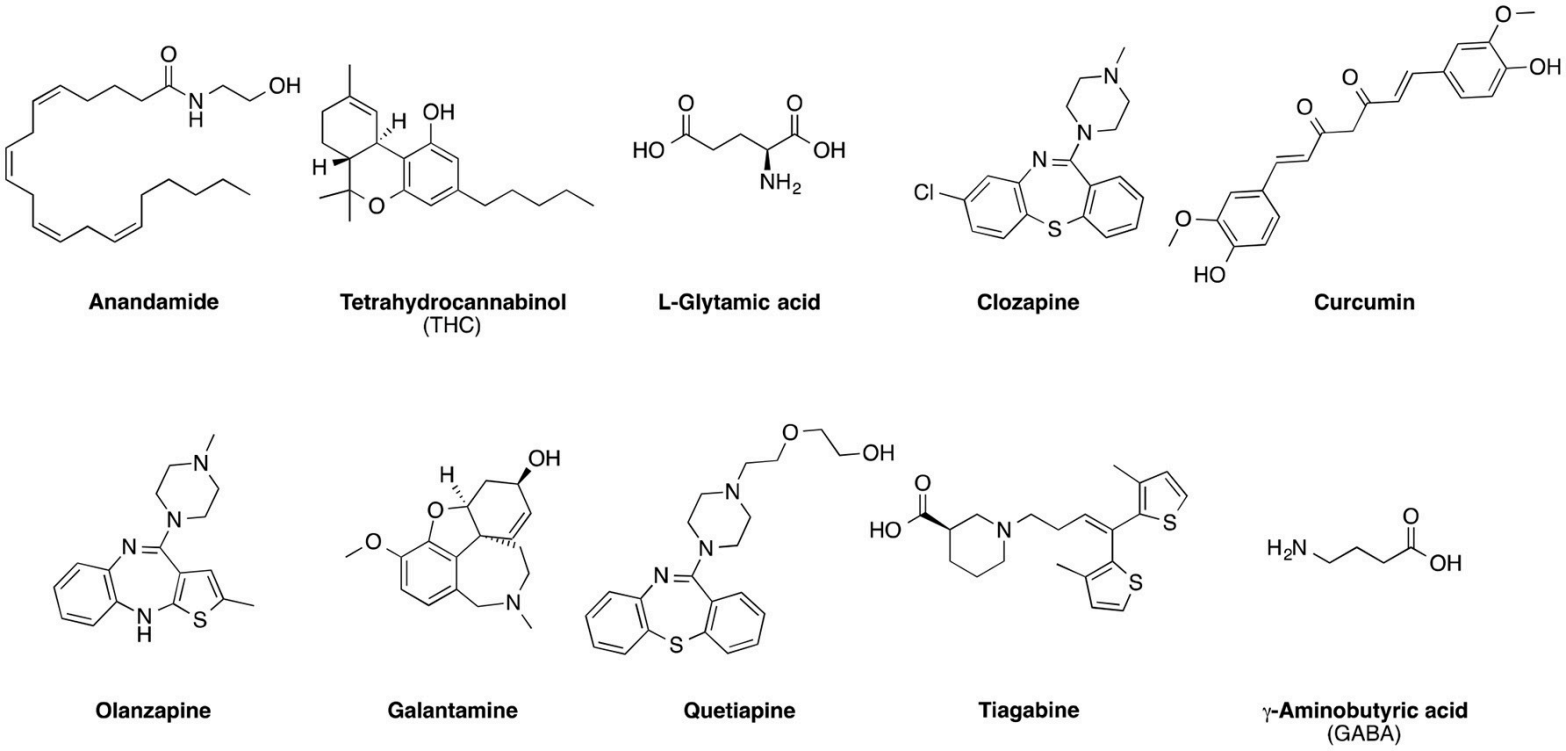
Chemical structures of selected pharmaceuticals and naturally occurring molecules, which were identified as recurring themes for neuropharmacology studies.

Neuroscience (or neurobiology) is a multidisciplinary scientific study of the nervous system, and especially its major organ, the brain. It was reported that neuropsychiatric disorders, together with brain connectivity and emotion, belong to the most cited works in neuroscience (Yeung et al., 2017a). Alzheimer's disease (AD), Parkinson's disease (PD), and related dementias are the most prevalent, aging-related neurodegenerative disorders of the central nervous system (CNS), affecting more than 17 (Reitz and Mayeux, 2014) and 21 million (Poewe et al., 2017) people worldwide, respectively. As a result of the global trend in population aging, the number of patients with AD, PD, and related dementias is projected to double over the next 20 years (Tzvetkov and Atanasov, 2018). These neuropsychiatric disorders represent a significant socioeconomic burden on society, and therefore, they have been heavily investigated and cited in the last few decades (Yeung et al., 2017a,c). Historically, clinical pharmacologists have worked with neuroscientists to develop human models in order to evaluate the translational value of selected drugs or drug receptors (biological targets) into clinical therapy (Trist et al., 2014). For example, the pharmacodynamics, pharmacokinetic, and metabolism of cannabinoids have been studied over three decades (Howlett et al., 2004). In Figure 1 are illustrated the chemical structures of the prominent cannabinoid tetrahydrocannabinol (THC) and other naturally occurring molecules and pharmaceuticals that were identified in this work as recurring theme for neuropharmacology studies. It is also worth mentioning that in the last decade the high publication rate of scientific literature on the field of neuropsychiatry correlates with the increased burden of neuropsychiatric disorders on society (Agarwal and Searls, 2009). Unfortunately, it seems that in recent years many leading pharmaceutical companies have reduced their investment in early drug discovery related to neuropsychiatric disorders (Trist et al., 2014). However, we strongly believe that it is important to further research on new neuropharmacology therapies for these neuropsychiatric disorders since a previous study has reported a startling 0.4% overall success rate of 413 clinical trials on AD conducted during 2002–2012 (Cummings et al., 2014).

To evaluate the impact of the cross-road of pharmacology and neuroscience and identify prevailing research topics in this area, a bibliometric analysis of the neuropharmacology field may allow for a deeper understanding of the current research hot topics and their citation performance in the scientific community. To the best of our knowledge, numerous bibliometric literature analyses have so far only touched upon a few aspects of the area of neuropharmacology, such as the distribution of author nationality and research topics in a selected neuropharmacology journal (Krishna Reddy et al., 2005), and the contribution of psychology researchers to psychopharmacology (Portillo-Salido, 2010). Therefore, the aim of this study is to identify and analyze the published neuropharmacology articles in the existing literature sources worldwide. The particular main goals of this work are:

1. To reveal and quantitatively compare the relevant key research topics in neuropharmacology as indicated by their citations,
2. To identify the institutions and countries having major contributions to this research area, and
3. To reveal and quantitatively compare which chemicals/pharmaceuticals have been major subjects of research in the area of neuropharmacology, and, respectively, received high citation counts.

## Materials and methods

### Data source

In April 2018, a comprehensive search was conducted using the Web of Science (WoS) Core Collection database, a multidisciplinary online database hosted by Clarivate Analytics, to identify manuscripts with the following search strategy: TOPIC = (“neuro^*^” AND “pharma^*^” AND (“compound^*^” OR “drug^*^” OR “molecule^*^”)). This strategy searched for manuscripts that contain the pre-defined combinations of these terms in their title, abstract, or keywords. No restrictions were placed on the publication year, manuscript type (e.g., research article, review, editorial, etc.), or publication language.

### Data extraction

The manuscripts resulting from the literature search were evaluated and recorded for: (1) publication year; (2) journal title; (3) total citation count; (4) authorship; (5) WoS category; and (6) manuscript type. The full records and cited references of these manuscripts were imported into VOSviewer for further bibliometric analyses, such as citation analysis of institutions, countries/regions, and journals.

VOSviewer extracts and analyzes the words in the titles and abstracts of manuscripts, relates them to citation counts, and finally, visualizes the results as a bubble map (Van Eck and Waltman, 2009). Each bubble represents a word or a phrase. We aimed to evaluate the field-specific words or phrases and thus excluded the top 5,000 common words from the Corpus of Contemporary American English (the list of words was obtained from https://www.wordfrequency.info/free.asp?s=y). Provided as a Supplementary Data Sheet, are all the 1,659 terms, remaining after the exclusion of the 5,000 common words, and their citations per manuscript. The bubble size indicates the frequency of occurrence of the words (multiple appearances in a single manuscript count as one). The bubble color indicates the averaged citation count received by manuscripts containing the word in their titles or abstracts. Two bubbles are in closer proximity if the two words had more frequent co-occurrence. The term map visualizes terms that appeared in at least 100 of the included manuscripts.

We tested for possible correlation between the total publication count and the averaged citations per manuscript for each institution, country/region, and journal that has reached the abovementioned threshold of having at least 100 manuscripts. Pearson's correlation test was performed in SPSS 24.0 (IBM, New York, NY, United States). Test results were considered significant if *p* < 0.05.

To better account for the potential confound of publication date on the citation count, we have also analyzed the normalized citation (NC) counts, which are expressed with values starting from 0 with no upper limit. When a term has NC = 1, it means that on average the publications in which the term occurs (in the title or abstract) have received the same number of citations as the average number of citations of all neuropharmacology publications (within the data set) from the same period (the same year of publication). Similarly, if NC = 2, the term has twice the number of citations than the average citation count of the publications from the same period. This corresponds to the concept of average NC impact in the VOSviewer.

## Results and discussion

**Figure 2:**
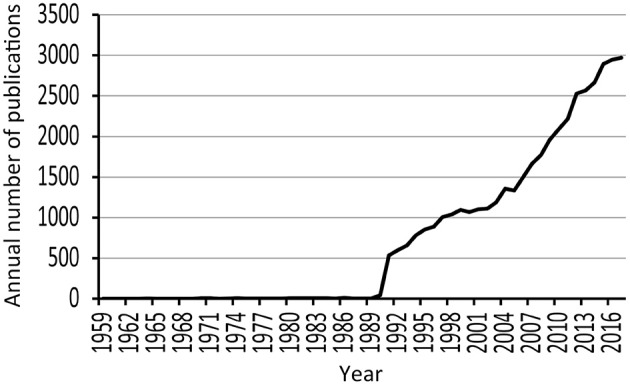
Publication trend of neuropharmacology manuscripts highlighting the continuous growth that has occurred since the 1990s.

The literature search resulted in 43,354 manuscripts. Figure 2 highlights the continuous growth of research in the area that has occurred since the 1990s. The small number of publications before the 1990s is likely due to absent indexing of older manuscripts (prior to 1990s) by the online literature database. The majority of the manuscripts were original articles (*n* = 31,360) and reviews (*n* = 11,266). The remaining manuscripts included proceedings papers (*n* = 2,124), editorial materials (*n* = 385), etc. Most of the manuscripts were written in English (*n* = 41,527, 95.8%). The top five WoS categories associated with the analyzed manuscripts were Pharmacology/Pharmacy (*n* = 14,892, 34.3%), Neurosciences (*n* = 11,747, 27.1%), Clinical Neurology (*n* = 4,981, 11.5%), Psychiatry (*n* = 4,464, 10.3%), and Biochemistry/Molecular Biology (*n* = 4,337, 10.0%). The manuscripts were contributed by over 100,000 authors from 15,890 organizations in 161 countries/regions and published in 4,374 journals.

Seven of the top ten most prolific institutions are located in the USA, and one each in Canada, Italy and the UK, respectively (Table 1). These institutions had 37.7–66.6 citations per manuscript. Meanwhile, the USA has accounted for 38% of the total number of publications (Table 2) and the top 10 most prolific countries/regions have 14.1–44.3 citations per manuscript. NC data has shown that manuscripts contributed by the top 10 most prolific institutions generally have had higher than average citations. However, this was not applicable to the top 10 countries/regions. The large volume of contributions from the USA, Germany, England, and China is consistent with previous analyses pointing their contributions to neuroimaging (Yeung et al., 2017b; Yeung, 2018), ethnopharmacology (Yeung et al., 2018a), and nutraceutical studies (Yeung et al., 2018b). Most of the top ten most prolific journals were those dealing either with neuroscience or pharmacology (Table 3). The multidisciplinary journal PLOS One had fewer citations per manuscript (16.3)—at most one-half of the other nine journals (32.7–77.3). One potential confound for this finding was that, for example, the peer-reviewed journal PLOS One was just recently founded (in 2006), and therefore papers published in it might have had less time to accumulate more citations. In this line, when NC was considered, manuscripts published in PLOS One were only slightly less cited than the average manuscripts published from the same year of publication (0.8, i.e., 20% less than the average). Pearson's correlation test revealed that there was significant correlation between total publication count and averaged citations per manuscript in the institution level (*r* = 0.39, *p* < 0.001), country level (*r* = 0.31, *p* = 0.043), but not the journal level (*r* = 0.15, *p* = 0.209).

**Table 1:** The top 10 contributing institutions.

**Contributor**	**Number of manuscripts (% of total)**	**Citations per manuscript**	**Normalized citation**
Harvard University	625 (1.4%)	66.6	1.9
University of Toronto	510 (1.2%)	55.4	1.6
University of Pennsylvania	403 (0.9%)	61.1	1.6
University of California San Francisco	402 (0.9%)	65.0	2.0
Johns Hopkins University	373 (0.9%)	48.9	1.7
University of California Los Angeles	365 (0.8%)	57.8	1.6
Yale University	361 (0.8%)	63.1	1.7
University of Pittsburgh	345 (0.8%)	46.0	1.5
University College London	329 (0.8%)	48.0	1.6
University of Milan	327 (0.8%)	37.7	1.3

**Table 2:** The top 10 contributing countries/regions.

**Contributor**	**Number of manuscripts (% of total)**	**Citations per manuscript**	**Normalized citation**
USA	16,461 (38.0%)	43.4	1.3
Germany	3,760 (8.7%)	34.2	1.1
Italy	3,623 (8.4%)	30.6	1.1
England	3,277 (7.6%)	43.3	1.3
France	2,716 (6.3%)	35.2	1.1
Canada	2,038 (4.7%)	44.3	1.3
China	2,031 (4.7%)	14.1	0.8
Japan	1,815 (4.2%)	28.4	0.8
Spain	1,770 (4.1%)	25.5	1.0
Australia	1,207 (2.8%)	28.7	1.2

**Table 3:** The top 10 contributing journals.

**Contributor**	**Number of manuscripts (% of total)**	**Citations per manuscript**	**Normalized citation**
Journal of Pharmacology and Experimental Therapeutics	664 (1.5%)	53.9	1.1
Neuropharmacology	645 (1.5%)	34.8	1.2
European Journal of Pharmacology	635 (1.5%)	32.8	0.8
British Journal of Pharmacology	579 (1.3%)	40.7	1.2
Journal of Neuroscience	577 (1.3%)	77.3	1.9
Journal of Medicinal Chemistry	515 (1.2%)	32.7	1.0
Psychopharmacology	513 (1.2%)	51.0	1.2
PLOS One^*^	463 (1.1%)	16.3	0.8
Neuroscience	421 (1.0%)	41.3	1.0
Neuropsychopharmacology	399 (0.9%)	59.6	1.7

* *Since 2006*.

**Figure 3:**
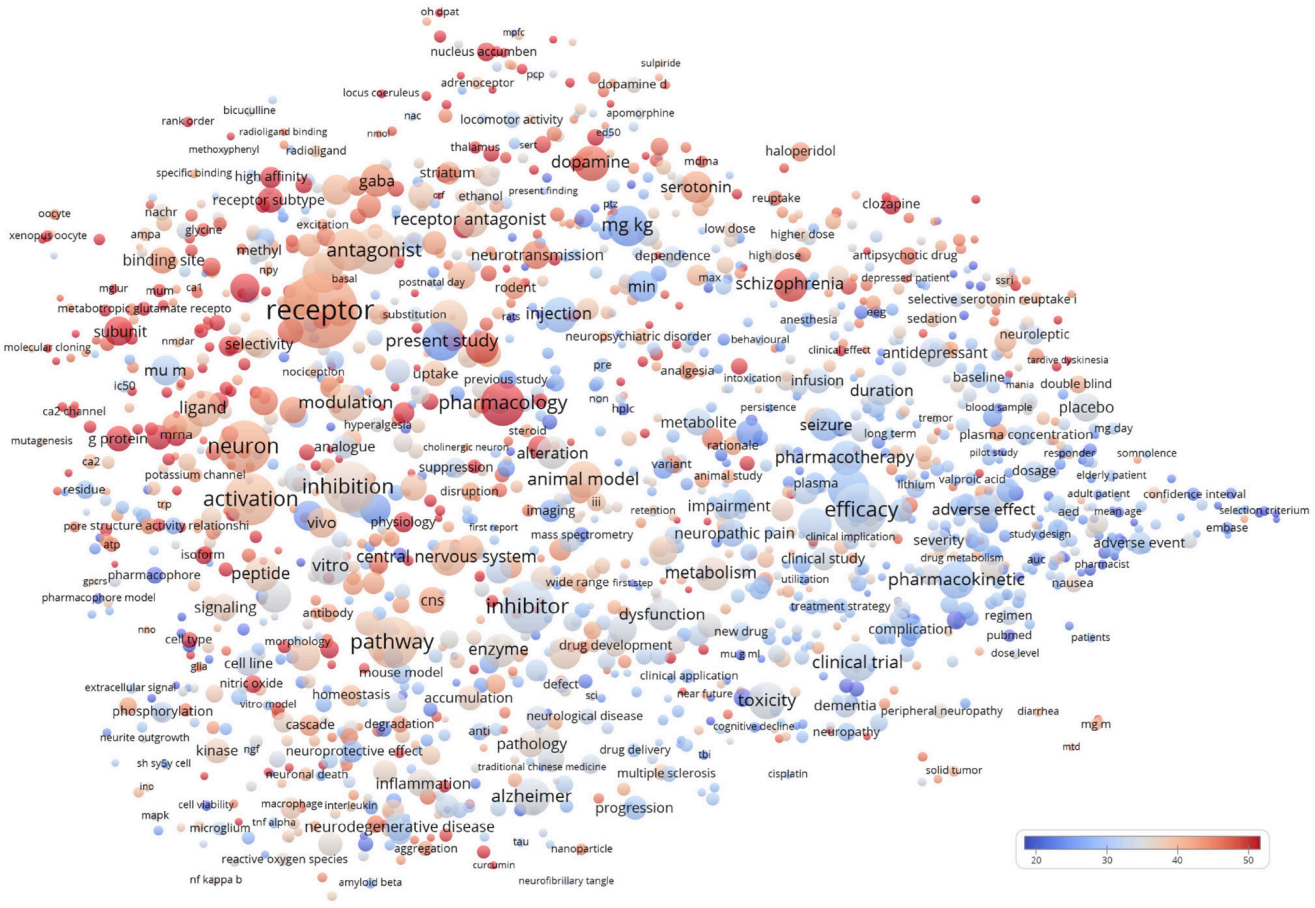
Term map using words from titles and abstracts of the 43,354 neuropharmacology articles. Words from titles and abstracts were parsed, analyzed and visualized by VOSviewer. There were 1,659 terms that appeared in 100 or more articles and hence are included in the term map. Each bubble represents a term or phrase. The bubble size indicates its frequency of occurrence. The bubble color indicates the averaged citation counts received by articles containing the term or phrase. If two terms co-occurred more frequently, the two bubbles would be in closer proximity.

By analyzing the words in the titles and abstracts of the 43,354 manuscripts, a bubble map was generated to visualize the citation data (Figure 3). There were 2,276 terms that appeared in at least 100 of the included manuscripts. After excluding words from the 5,000 common word list (from the Corpus of Contemporary American English; https://www.wordfrequency.info/free.asp?s=y), 1,659 terms remained (provided as a Supplementary Data Sheet, are all the 1,659 terms and their citations per manuscript). Differential scientific attention has been given to several pathological conditions. For instance, schizophrenia (2,093 manuscripts; 48.1 citations per manuscript; NC = 1.3) had higher citations per manuscript than PD (1,854 manuscripts; 37.0 citations per manuscript; NC = 1.2), autism (159 manuscripts; 34.4 citations per manuscript; NC = 1.6), and AD (2,506 manuscripts; 34.2 citations per manuscript; NC = 1.2), which in turn had higher citations than neuropathic pain (1,349 manuscripts; 30.0 citations per manuscript; NC = 1.0) and seizure (1,915 manuscripts; 29.2 citations per manuscript; NC = 0.9). Interestingly, AD and autism have been identified as “hot topics” in the general neuroscience research field with continuously increasing citations rate during the recent years (2006–2015) (Yeung et al., 2017c). However, the current results confirm that other neuropsychiatric disorders (e.g., schizophrenia and PD) have also attracted big attention in the specific field of neuropharmacology. Meanwhile, manuscripts dealing with activation or inhibition pathways of receptors in neurons had both higher publication count and citations per manuscript than those dealing with adverse effect or adverse event of neuropharmacological drugs. This might be due to the universal and far-reaching relevance of molecular regulatory mechanisms characterization (receptor modulation, inhibition, or activation of pathways) as compared adverse effects/events reports that might be resulting in diminishing in the interest in the therapies that exhibited it.

Manuscripts mentioning certain chemicals or pharmaceuticals displayed high citations per manuscript, such as those reporting about anandamide, THC, L-glutamate, clozapine, and curcumin (Figure 1 and Table 4). The chemicals or pharmaceuticals with at least 50 citations per manuscript were mostly related to cannabis and anti-psychotic drugs, as well as some dealing with anti-epilepsy or AD (Table 4). Cannabis and cannabinoids have long been known to exert multiple effects on the CNS, cardiorespiratory system, eye, immune system, and reproductive system (Pertwee, 2000; Kumar et al., 2001). Perhaps the most important effect associated with cannabis and cannabinoids is their use as analgesics (Miller and Miller, 2017). Cannabis pharmacology has firstly attracted the attention in the 1960s. Nowadays, researchers have been also exploring its use in the treatment of psychiatric disorders and cancer (Russo and Marcu, 2017). Besides, two natural products, curcumin and galantamine, were on the list of the most mentioned molecules. It was also reported that curcumin and its derivatives have anti-inflammatory properties that may have a potential role in treating neurodegenerative diseases such as AD and PD (Lee et al., 2013). Meanwhile, galantamine is used to treat AD as it has the effects of blocking acetylcholinesterase and modulating nicotinic acetylcholine receptors (Arias et al., 2004). It is worth noting that galantamine is the only current drug of plant origin against dementia (Tewari et al., 2018). Continuous pre-clinical research efforts are also focused on neuropharmacology effects mediated from a range of other natural products derived from medicinal plants or dietary sources (Baur et al., 2014; Ajami et al., 2017; Khan et al., 2017; Nabavi et al., 2018; Valenti et al., 2018). The presence of natural products as a key theme in a large volume of neuropharmacology manuscripts should come as no surprise, taking into consideration the importance of natural product pharmacology, both historically as well as in modern drug discovery (Wang et al., 2014; Atanasov et al., 2015; Waltenberger et al., 2016; Uhrin et al., 2018).

**Table 4:** Chemicals and pharmaceuticals that had at least 50 citations per manuscript.

**Contributor**	**Number of manuscripts (% of total)**	**Citations per manuscript**	**Normalized citation**
Anandamide	214 (0.5%)	80.1	1.8
Tetrahydrocannabinol	212 (0.5%)	72.6	1.9
L-Glutamate	132 (0.3%)	71.6	1.4
Clozapine	676 (1.6%)	59.7	1.2
Curcumin	118 (0.3%)	58.2	2.5
Olanzapine	213 (0.5%)	56.6	1.2
Galantamine	123 (0.3%)	56.4	1.7
Endocannabinoid	262 (0.6%)	55.3	1.6
Cannabinoid	523 (1.2%)	51.5	1.4
Quetiapine	166 (0.4%)	50.9	1.3
Tiagabine	126 (0.3%)	50.7	1.1
Gamma aminobutyric acid	625 (1.4%)	50.0	1.1

While this bibliometric analysis outlines the prevailing publication and citation trends of the neuropharmacology field, it should be noted that the choice of the keywords had an influence on the body of literature that was identified and analyzed, and therefore, all relevant neuropharmacology manuscripts could not be covered. For example, while our analysis includes manuscripts mentioning “neuro^*^” AND “pharma^*^” AND “compound^*^” manuscripts mentioning “neuro^*^” in combination with a specific drug name (e.g., “neuro^*^” AND “clozapine”) might not necessarily be included (depending on the entire sets of terms used in the respective manuscripts). Another important limitation for readers to consider as they interpret the presented data of citation per manuscript is that a few very highly cited articles might have skewed the results, and geometric or truncated means should perform better to account for that. However, using geometric or truncated means with VOSviewer is not possible. With consideration of this, in the main text and tables we have only listed the terms that have appeared in at least 100 manuscripts. Besides, we report NC that account for citation performance in comparison to the average number of citations of all neuropharmacology publications (within the data set) from the same period (the same year of publication).

In conclusion, a bibliometric analysis was performed to evaluate manuscripts focused on neuropharmacology. The obtained data indicated that the USA is the major contributor to this research field and seven of the top 10 most prolific institutions are located in the USA. The most prolific journals were mainly specialized in neuropharmacology. Manuscripts involving cannabis, cannabinoid, antipsychotic drugs, anti-epileptic drugs, and curcumin had more than 50 citations per manuscript. Manuscripts dealing with schizophrenia and AD also displayed a substantial number of citations per manuscript. With virtually all of the articles published since the 1990s, the research area of neuropharmacology has been growing steadily. With the ever-growing neuroscience research, it is hoped that new pharmaceutical molecules or new therapeutic applications of discovered molecules will continue to be introduced and will continuously contribute for the future improvement of patient's health and well-being.

## Author contributions

AY and AA conceived the work. AY acquired data and drafted the work. AY and AA analyzed data. AA and NT critically revised the work. All authors have approved the final content of the manuscript.

### Conflict of interest statement

The authors declare that the research was conducted in the absence of any commercial or financial relationships that could be construed as a potential conflict of interest.

## Supplementary material

Supplementary data: A supplementary data sheet (XLSX) includes 1,659 terms that occurred in at least 100 of all studied 43,354 manuscripts and their citations per manuscript.
